# Effects of monocyte chemoattractant protein‐1, macrophage inflammatory protein‐1α, and interferon‐α2a on P450 enzymes in human hepatocytes in vitro

**DOI:** 10.1002/prp2.551

**Published:** 2019-12-10

**Authors:** Maciej Czerwiński, Krystal Gilligan, Kevin Westland, Brian W. Ogilvie

**Affiliations:** ^1^ Sekisui XenoTech, LLC Kansas City KS USA

**Keywords:** chemokine, CYP enzymes, cytokine, interferon‐alpha

## Abstract

Some immunomodulatory agents stimulate the release of cytokines capable of suppressing P450 enzymes and potentially affecting pharmacokinetics of coadministered medications. Cytokines released in response to an immunomodulator in the blood ex vivo can be used to screen for the potential for drug‐drug interactions. Tilsotolimod, an investigational agonist of Toll‐like receptor 9, stimulated the release of macrophage chemoattractant protein‐1 (MCP‐1), macrophage inflammatory protein‐1α (MIP‐1α), and interferon‐α2a (INF‐α2a) in blood obtained from healthy donors. Although tilsotolimod did not directly affect CYP1A2, CYP2B6, or CYP3A4 expression or activity, the cytokines stimulated by the drug reduced CYP1A2 and CYP2B6 enzyme activities in cultured human hepatocytes. This study sought to identify which cytokines were responsible for tilsotolimod's indirect effects on P450 enzymes in vitro. A 72‐h treatment with recombinant human chemokines MCP‐1 and MIP‐1α did not alter CYP1A2, CYP2B6, CYP2C8, CYP2C9, CYP3A4, or signal transducer and activator of transcription 1 (STAT1) mRNA expression or CYP1A2, CYP2B6, or CYP3A4/5 enzyme activity in cocultures of human hepatocytes and Kupffer cells. INF‐α2a, at 2.5 ng/mL but not at the lower concentrations applied to the cells, increased CYP1A2 and STAT1 mRNA by 2.4‐ and 5.2‐fold, respectively, and reduced CYP2B6 enzyme activity to 46% of control. This study established that INF‐α2a, but not MCP‐1 or MIP‐1α, mediated tilsotolimod effects on CYP1A2 and CYP2B6 expression in human hepatocytes.

**Abbreviations**
IL: interleukin
INF: interferon
LPS: lipopolysaccharide
MCP‐1: macrophage chemoattractant protein‐1
MIP‐1α: macrophage inflammatory protein‐1α
P450: cytochrome P450
STAT1: signal transducer and activator of transcription 1
TLR: Toll‐like receptor
TNF‐α: tumor necrosis factor‐α

## 1 INTRODUCTION

Some immunomodulatory drugs stimulate the innate immune system to release the cytokines which may change the expression of drug metabolizing enzymes. In drug development, regulation of mutiple pro‐ and anti‐inflammatory cytokines by Toll‐like receptors (TLR) has gained attention in parallel to targetting the therpapeutic potential of these receptors.^1^, ^2^ In a previous study, tilsotolimod, an agonist of TLR9 designed to enhance T‐cell responses to tumor antigens, stimulated the release of cytokines in human blood and the effects of these cytokines on P450 enzymes in human hepatocytes treated for 72 hours were measured in vitro.^3^ Tarantino and co‐authors (2018) demonstrated that the treatment of blood with tilsotolimod (10 µg/mL) resulted in significant elevation of interferon gamma‐induced protein‐10 (IP‐10) and macrophage chemoattractant protein‐1 (MCP‐1), while a higher concentration (100 µg/mL) significantly increased macrophage inflammatory protein‐1α (MIP‐1α), tumor necrosis factor‐α (TNF‐α), and interferon‐α2a (INF‐α2a) over control. The cytokines contained in plasma isolated from whole blood treated with tilsotolimod (100 µg/mL) reduced CYP1A2 and CYP2B6 enzyme activity to 47% and 13% of control, respectively, in human hepatocytes. In the same study, bacterial lipopolysaccharide (LPS), an agonist of TLR4, significantly elevated IP‐10, MIP‐1α, and pro‐inflammatory IL‐2, IL‐6, IL‐12p70, and TNF‐α but not MCP‐1 or INF‐α2a. MCP‐1 and IFN‐α2a were increased 7.2‐ and 1500‐fold, respectively, in the tilsotolimod‐treated plasma over the LPS‐treated plasma. The LPS‐treated plasma suppressed the CYP1A2 and CYP2B6 enzymatic activity by 79% and 84%, respectively.^3^ The aim of this study was to establish which of the tilsotolimod‐stimulated cytokines were responsible for the suppression of CYP1A2 and 2B6. Cocultures of hepatocytes and Kupffer cells were treated with the recombinant human MCP‐1, MIP‐1α, and IFN‐α2a followed by the measurement of CYP1A2, CYP2B6, CYP2C8, CYP2C9, CYP3A4, and signal transducer and activator of transcription 1 (STAT1) mRNA expression and enzyme activity of CYP1A2, CYP2B6, and CYP3A4/5. The evidence is provided that IFN‐α2a increased CYP1A2 and STAT1 mRNA and reduced CYP2B6 enzyme activity, in human hepatocytes in vitro. These data may assist in evaluating the potential of immunomodulatory drugs to modify P450 expression in vivo.

## 2 MATERIAL AND METHODS

### 2.1 Chemicals and reagents

Interleukin (IL)‐6 was purchased from Thermo Fisher (cat. # PHC0064, lot 1898414) and reconstituted in 100 mmol/L acetic acid. MCP‐1 (cat. # M6667, lot MKCF8074) and INF‐α2a (cat. # SRP4594, lot 4B08L45940) were purchased from Sigma Millipore. MIP‐1α (cat. # 270‐LD/CF, lot CG1217092) was purchased from R&D Systems. Normal plasma used in hepatocyte cultures was donated by three healthy volunteers, two men and one woman, following informed consent to participate in the study. The TaqMan assays (Thermo Fisher Scientific) used in this study were Hs00167927_m1 (CYP1A2), Hs03044634_m1 (CYP2B6), Hs00258314_m1 (CYP2C8), Hs00426397_m1 (CYP2C9), Hs00604506_m1 (CYP3A4), Hs00234829_m1 (STAT1), Hs99999905_m1 (GAPDH), and Hs99999901_s1 (18S). The sources of the other reagents used in this study have been described elsewhere.^4^

### 2.2 Cell culture and treatments, mRNA and enzyme activity analysis

The cell culture procedures and treatments as well as the analysis of mRNA and enzyme activities were conducted as recently described.^5^ Demographic data of liver donors, two men and one woman, are provided in Table 1. The modified Chee's medium was supplemented with 10% normal pooled human plasma in order to replicate conditions of the initial in vitro evaluation of tilsotolimod. Hepatocytes were treated with MCP‐1 (2, 10 or 50 ng/mL), MIP‐1α (0.4, 2 or 10 ng/mL), and INF‐α2a (0.1, 0.5, or 2.5 ng/mL) with dosing solutions prepared fresh daily for each of 3 days of treatments. These concentrations were 0.2‐, 1‐, or 5‐fold the concentration found in plasma stimulated with tilsotolimod (100 µg/mL).^3^ The levels of STAT1 mRNA were evaluated only for treatments with IL‐6, INF‐α2a, and phenobarbital. Representative cultures were examined daily with light microscopy for signs of treatment toxicity such as cell membrane deterioration, swollen nuclei, or increased number of cytoplasmic vacuoles. Differences between treatment and control groups were analyzed with one‐tailed *t* test (SigmaPlot^™^ 12.5, Systat Software, Inc).

**Table 1:** Liver donor information

XT #	Gender	Age (years)	Ethnicity	BMI	Tobacco use	Alcohol use	Cause of death
H1281	M	61	Caucasian	21.0	No	1‐3 drinks on weekends^a^	Anoxia
H1408	F	19	Caucasian	29.5	No	No	Head trauma
H1412	M	44	Caucasian	34.6	No	No	Anoxia

a Information provided by the Organ Procurement Organization, as reported by the next of kin.

## 3 RESULTS

### 3.1 Effects of MCP‐1, MIP‐1α, or INF‐α2a on P450 mRNA expression in human hepatocytes

**Figure 1:**
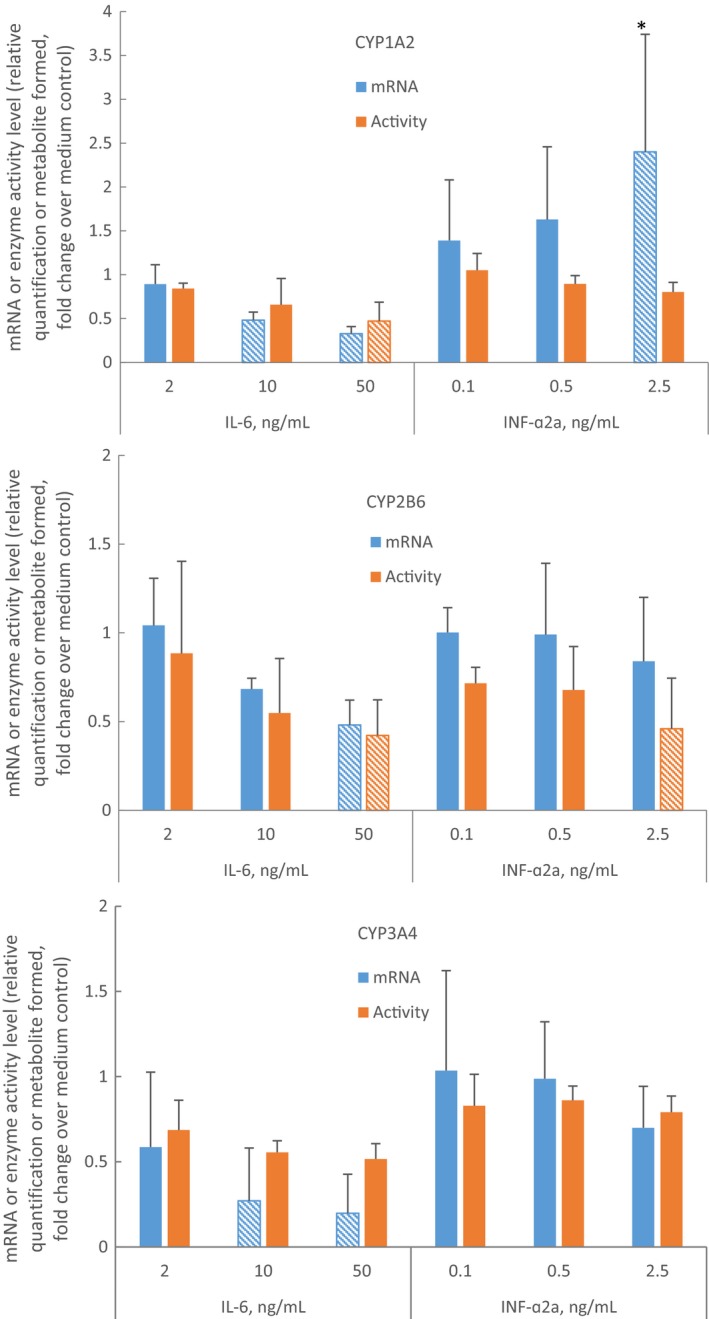
Effects of IL‐6 and INF‐α2a on CYP1A2, CYP2B6, and CYP3A4 mRNA expression and enzyme activity in culture of primary human hepatocytes. Hepatocyte‐Kupffer cell cocultures (N = 3, experiment in cells from each donor was conducted once) were incubated for 72 hour with medium alone, medium containing phenobarbital (750 µmol/L), IL‐6 (2, 10 or 50 ng/mL), or INF‐α2a (0.1, 0.5 or 2.5 ng/mL). The mRNA levels of each P450 were quantitated by qPCR with normalization to glyceraldehyde 3‐phosphate dehydrogenase mRNA and to the mRNA levels of the P450 in the medium control cultures. The enzymatic activity of the P450 enzymes were determined as described in Materials and Methods. Data were normalized to medium control cultures values set to equal 1. Patterned bars indicate observations of mRNA or enzyme activity levels below 50% of the control or observations of enzyme activity above 2‐fold of the control. Asterisk (*) indicates difference from the control with one‐tailed *t* test, *P* < .05 (SigmaPlot™ 12.5, Systat Software, Inc)

**Figure 2:**
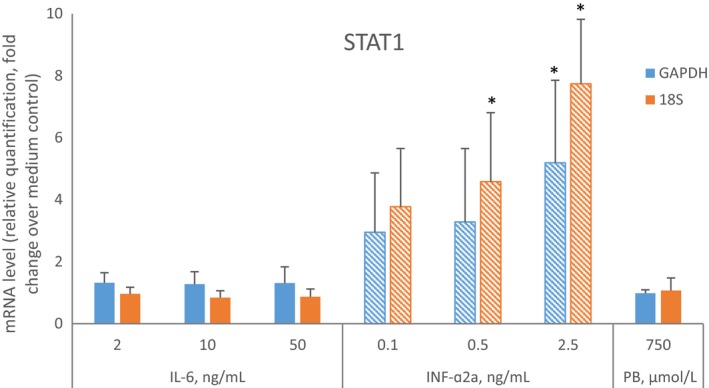
Effects of IL‐6, INF‐α2a, and phenobarbital (PB) on STAT1 mRNA expression in primary human hepatocytes. The hepatocyte‐Kupffer cell cocultures used to determine levels of P450 enzymes (N = 3, experiment in cells from each donor was conducted once) were incubated for 72 hour with medium alone, medium containing IL‐6 (2, 10, or 50 ng/mL), INF‐α2a (0.1, 0.5, or 2.5 ng/mL), or PB (750 µmol/L). The mRNA levels were quantitated by qPCR with normalization to glyceraldehyde 3‐phosphate dehydrogenase or 18S mRNA and to the mRNA levels of the STAT1 in the medium control cultures. The normalization to 18S mRNA was performed to match conditions used by Chen et al.^6^ Data were normalized to medium control cultures values set to equal 1. Patterned bars indicate mRNA above 2‐fold of the control. Asterisks (*) indicate difference from the control with one‐tailed *t* test, *P* < .05 (SigmaPlot™ 12.5, Systat Software, Inc)

For brevity, only the average reduction to less than 50% or the increase of at least 2‐fold over the medium control in cultured cells from three donors were reported. Figure 1 illustrates all effects of IL‐6 and INF‐α2a on CYP1A2, CYP2B6, and CYP3A4 mRNA and the enzyme activity. Phenobarbital (750 µmol/L), a positive control for induction through nuclear constitutive androstane receptor or pregnane X receptor pathways, increased the mRNA expression of CYP2B6 (5.3‐fold), CYP2C8 (2.3‐fold), CYP2C9 (3.6‐fold), and CYP3A4 mRNA (26.1‐fold) over control. IL‐6 (10 or 50 ng/mL), a positive control for suppression by a cytokine, reduced mRNA expression of CYP1A2, CYP2B6, CYP2C9, and CYP3A4 mRNAs, to 33%, 48%, 49% and 20% of control, respectively. MCP‐1 or MIP‐1α did not have an effect on the mRNA expression of any of the enzymes evaluated. INF‐α2a (2.5 ng/mL) increased CYP1A2 mRNA expression 2.4‐fold over control. INF‐α2a (0.1, 0.5, and 2.5 ng/mL) increased STAT1 mRNA expression in a concentration‐dependent manner (2.9‐, 3.3‐, and 5.2‐fold over control, respectively). IL‐6 (2, 10, or 50 ng/mL) or phenobarbital (750 µmol/L) did not have an effect on STAT1 mRNA expression (Figure 2). Microscopic examination of representative cultures did not reveal signs of toxicity of the treatments.

### 3.2 Effects of MCP‐1, MIP‐1α, or INF‐α2a on P450 enzyme activity in human hepatocytes

Phenobarbital increased CYP1A2 activity by 2.3‐fold, CYP2B6 activity by 7.8‐fold, and CYP3A4/5 activity by 15.6‐fold. Figure 1 shows that IL‐6 (50 ng/mL) reduced CYP1A2 and CYP2B6 enzyme activities to 47% and 42% of control, respectively. MCP‐1 or MIP‐1α did not have effect on CYP1A2, CYP2B6, or CYP3A4/5 enzyme activity. INF‐α2a (2.5 ng/mL) reduced CYP2B6 enzyme activity to 46% of control (Figure 1).

## 4 DISCUSSION

In this study, recombinant INF‐α2a induced CYP1A2 mRNA 2.4‐fold in cultured hepatocytes, consistent with the 1.5‐fold increase caused by plasma from tilsotolimod‐treated blood.^3^ While INF‐α2a increased CYP1A2 mRNA levels, it caused an unexpected, but statistically insignificant, decrease in enzyme activity. A similar discrepancy between increased CYP1A2 mRNA and decreased enzyme activity levels was observed in hepatocytes cultured with plasma from tilsotolimod‐treated blood. In this study, recombinant INF‐α2a decreased CYP2B6 enzyme activity, consistent with the effects of plasma from tilsotolimod‐treated blood on the enzyme in plated hepatocytes.^3^ The effects of INF‐α2a on CYP1A2 and CYP2B6 were dose‐dependent and reached thresholds of 2‐fold increase or a 50% reduction at concentration of the cytokine that was 5‐fold higher than that found in the plasma from tilsotomod‐treated blood.

The effects of INF‐α2b, which is pharmacologically indistinguishable from its allelic variant INF‐α2a, on P450 enzymes in cocultures of human primary hepatocytes and non‐parenchymal liver cells were previously examined.^6^ Chen and coauthors reported that INF‐α2b (0.1‐10 ng/mL) induced CYP3A4 mRNA and protein. It is suspected that the differences in handling of the cells contributed to the discrepant CYP3A4 results from the two studies. The cultures utilized by Chen et al were prepared by a hepatocyte vendor and shipped to the testing laboratory overnight in preservation buffer at 4°C. Upon receipt, preservation buffer was replaced with Williams’ E culture medium and cells were allowed a day for recovery and adaptation. The effects of the interferon on the mRNA or the protein were measured following drug treatment for 2 or 3 days, respectively. In the present study, the cells were isolated and the cocultures were maintained continuously in modified Chee's medium in an atmosphere of 5% CO_2_ at 37°C for 5 days in one laboratory. Both mRNA and enzyme activity were measured at a single time point, 72 hour post‐treatment. Analysis of STAT1 mRNA, the INF‐α target gene, demonstrated functionality of interferon receptor in the cultured cells in both studies, with a 5.2‐fold induction in this study and 7.6‐fold reported by Chen et al.^6^ The organ donors’ demographic data, causes of death, plating viability, and sandwich culture method utilizing type I collagen and Matrigel were similar between the two studies. However, it is possible that the CYP3A4 mRNA response to INF‐α2a was attenuated by the addition of normal plasma (10% v/v) to the cell culture medium in our study. In a previous study, we observed that the addition of plasma (10%, v/v) from blood incubated with saline (2% v/v, 24 hour) to cell culture medium for 72 hour suppressed CYP3A4 mRNA in hepatocytes from three donors.^5^

The absence of appreciable effects of recombinant INF‐α2a on CYP3A4 is consistent with the conclusion that interferon‐α can be coadministered with the drugs metabolized by CYP1A2 and CYP3A4 in patients with chronic hepatitis C without significant risks of drug interactions, although an evaluation of CYP2B6 was not provided.^7^ On the other hand, pegylated INF‐α2a PEGASYS inhibited P450 1A2 and increased theophylline total exposure by 25% in healthy subjects.^8^

The effects of MCP‐1 or MIP‐1α on P450 enzymes have not been reported, but since these chemokines are expected to be elevated by some drugs targeting TLRs, they will need to be considered from a drug safety perspective.^9^ It is a limitation of this study that cytokine‐stimulated differentiation of monocytes to macrophages could not be evaluated in vitro, as macrophages are a source of pro‐inflammatory cytokines.^10^

This study established that INF‐α2a, in a dose‐dependent manner, increased CYP1A2 mRNA and reduced CYP2B6 enzyme activity in human hepatocytes in vitro. We concluded that INF‐α2a, but not chemokines MCP‐1 or MIP‐1α, was likely responsible for appreciable P450 changes observed in hepatocytes cultured with plasma from tilsotolimod‐treated blood. It is possible that other cytokines modulated by tilsotolimod but not evaluated in this study, could also modify CYP enzymes mRNA expression or activity.

## DISCLOSURE

All the authors are employees of Sekisui XenoTech LLC and do not have a conflict of interest related to preparation and publication of this research article.

## AUTHOR CONTRIBUTIONS

Czerwiński participated in research design and performed data analysis. Green and Westland conducted the experiments. Czerwiński and Ogilvie wrote or contributed to the writing of the manuscript.
